# Designing Ordered Structure with Piezoceramic Actuation Units (OSPAU) for Generating Continual Nanostep Motion

**DOI:** 10.1002/advs.202001155

**Published:** 2020-07-02

**Authors:** Zhanmiao Li, Xiangyu Gao, Jikun Yang, Xudong Xin, Xingyu Yi, Lang Bian, Shuxiang Dong

**Affiliations:** ^1^ Department of Materials Science and Engineering College of Engineering Peking University Beijing 100871 China; ^2^ Beijing Key Laboratory for Magnetoelectric Materials and Devices (BKL‐MEMD) Beijing 100871 China

**Keywords:** artificially generated quasi shear mode (AGQSM), cofired multilayer ceramic, nanostep motion, ordered structure with piezoceramic actuation units (OSPAU), piezoelectric actuators

## Abstract

Continual precision actuations with nanoscale resolution over large ranges have extensive requirements in advanced intelligent manufacturing and precise surgical robots. To produce continual nanostep motion, conventionally, multiple pairs of piezo‐actuators are employed to operate in inchworm principle under complex three‐ or four‐phase timing signal drive. Inspired by the idea of ordered structures with functional units, a much simpler nanostep piezoelectric actuator consisting of (2 × 2) arrayed, cofired multilayer piezoceramic actuation units is developed, which operates in an artificially generated quasi shear mode (AGQSM) that is missing in natural piezoelectric ceramics. Under only one‐phase square‐wave voltage drive, the actuator can produce a stable, continual nanostep motion in two ways at nonresonant frequencies, and the obtained minimum step displacement is as low as 7 nm in open control, indicating its potential application as a precise finger or knife actuator in surgical robots. This work is of great guiding significance for future actuator designs using the methodology of ordered structure with piezoceramic actuation units and AGQSM.

## Graphical abstract

According to the methodology of ordered structure with piezoceramic actuation units, a (2 × 2) arrayed, cofired multilayer piezoceramic actuator operating in an artificially designed quasi‐d_34_ shear mode is built and validated for realizing continual step motion from nano‐ to macro‐scale. Its structure is much simpler than traditional nanostep actuators and obtained minimum step displacement is only 7 nm.

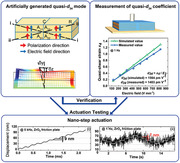

## 1 Introduction

Nanostep actuations are playing indispensable roles in microelectronic manufacturing like lithography mask aligner,^[^ ^1^, ^2^, ^3^, ^4^ ^]^ and minimally invasive surgical robot fields.^[^ ^5^, ^6^, ^7^, ^8^ ^]^ Although electromagnetic actuators are dominant force generation devices,^[^ ^9^, ^10^ ^]^ they are difficult to perform nanostep motion. With the rapid development in the high‐tech areas, there are great demands for piezoelectric actuators with the nanometer resolution to meet various precise positioning challenges.^[^ ^11^, ^12^ ^]^ Typically, the piezoelectric actuators can be classified into two types: i) resonant actuators, and ii) nonresonant actuators. The resonant piezoelectric motors usually use hybrid modes, including bending‐bending, longitudinal‐bending, and longitudinal‐torsional resonant vibration modes, etc., to drive a slider or rotor moving via high‐frequency frictional force.^[^ ^13^, ^14^, ^15^, ^16^ ^]^ The simultaneous excitation of hybrid modes normally needs the specific geometry structure design though the ultrasonic motors can produce relatively higher motion speed and larger driving force.^[^ ^17^, ^18^, ^19^ ^]^ Meanwhile, efforts have been devoted to single‐mode resonant ultrasonic motors for lower size requirements.^[^ ^20^, ^21^ ^]^ Nonetheless, the sensitivity of the resonant frequency to environment temperature change and the severe high‐frequency friction wears have restricted the moving stability, displacement resolution and positioning accuracy of the resonant actuators.

Piezoelectric nonresonant actuators are not sensitive to environment temperature change and work in a broad frequency‐band with much higher displacement resolution in comparison to resonant ultrasonic motors.^[^ ^22^, ^23^ ^]^ The nonresonant step actuators generally include i) the inertia type and ii) the inchworm type. Normally working in a single mode, inertia actuators achieve step motion using the friction force changes caused by inertia force when slow extending (or quick contracting) is produced.^[^ ^4^, ^24^ ^]^ But inevitably, inertia actuators always produce unwished and quite large backward motion in one‐step motion cycle due to the inertial force.^[^ ^25^ ^]^ As another typical type, the inchworm actuators are composed of at least one driving unit and two clamping units, imitating motion process of inchworms in nature.^[^ ^26^, ^27^ ^]^ Their systems ought to involve at least three independent actuating units to implement a series of clamping, feeding, and releasing motions, leading to quite large and complex structures.

As basic actuating units, the cofired multilayer piezoelectric actuators are widely used in various precision machines and manufacturing fields as they possess large generation force, compact sizes, low driving voltage, fast response, and superior integratability, even though they could produce only a simple longitudinal displacement in very limited travel range (typically, zero to tens micrometers).^[^ ^28^, ^29^ ^]^ Nonetheless, cofired multilayer actuators operating in the shear mode, which are necessary for generating transverse displacement, have never been fabricated before because the polarization direction of each ceramic layer is perpendicular other than parallel to its thickness direction, which causes the conflict between the poling direction and applied electric direction.^[^ ^30^, ^31^ ^]^ Traditionally, researchers or engineers have to use glue method instead of cofired method to fabricate d_15_ shear‐mode multilayer actuators, resulting in shear strain loss and low fabrication efficiency. In addition, the working voltage applied to one d_15_ multilayer actuator is limited to prevent domains 90° switching in ceramic layers caused by a high electric field, which may result in d_15_ coefficient to be zero. Recently, it was found that some piezoelectric single crystals, such as (1−*x*)Pb(Mg_1/3_Nb_2/3_)O_3_‐*x*PbTiO_3_ (PMN‐PT), have quite high d_36_ coefficient suitable for fabricating shear‐mode actuators,^[^ ^21^, ^32^, ^33^ ^]^ but the brittle character and expensive cost even over gold impeded their applications.

As a typical electromechanical coupling counterpart, the polarized Pb(Zr,Ti)O_3_ (PZT) piezoelectric ceramics feature the 6 mm point group and have only five nonzero piezoelectric strain coefficients including d_33_, d_31_ (d_32_), and d_15_ (d_24_), which have restricted the design and application of piezoelectric devices.^[^ ^34^, ^35^ ^]^ To break the bottleneck, it is necessary to develop neo‐type cofired multilayer piezoelectric ceramic and realize an artificially generated quasi shear mode (AGQSM) for nanoscale positioning. Inspired by the idea of ordered functional units, which pursues abnormal physical properties that are not existent in the original materials,^[^ ^36^, ^37^, ^38^, ^39^ ^]^ it is possible to design an ordered structure with piezoceramic actuation units (OSPAU) for artificially generating new strain modes, such as quasi‐d_34_ shear strain mode that is not existent in nature ceramics. The proposed methodology of OSPAU has potential to solve the puzzling problem mentioned above.

In this work, a (2 × 2) arrayed, cofired multilayer ceramic operating in quasi‐d_34_ shear mode was designed and fabricated. Moreover, a nonresonant nanostep piezoelectric actuator operating in quasi‐d_34_ mode, which is totally different from inchworm principle, nor an inertial force mechanism or ultrasonic high‐frequency resonant vibration mechanism, was developed for nano actuating in wide range. We will see that the proposed nonresonant piezoelectric actuator can produce continual nanostep motion with a minimum step as low as 7 nm in open‐loop control. The proposed methodology provides a novel perspective on expanding the applications of nano actuators in bioengineering field.

## 2 Results and Discussion

### 2.1 Designing Quasi‐d_34_ Shear Mode Based on OSPAU Method

**Figure 1:**
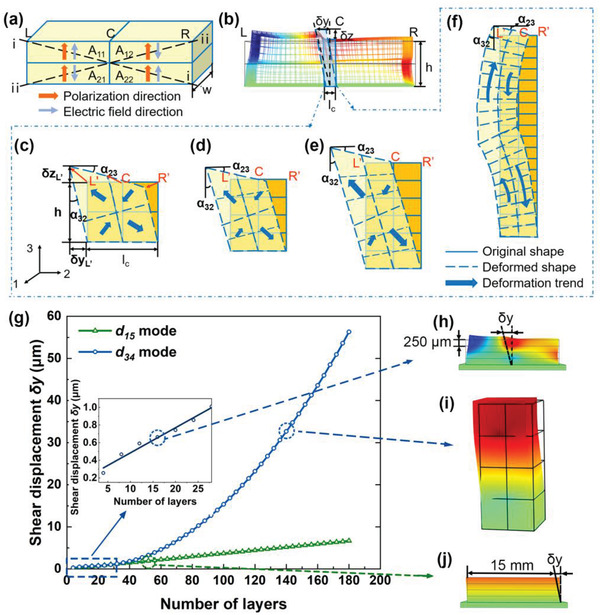
The quasi‐d_34_ shear mode analyses. a) The schematic diagram of the square multilayer ceramic with four subunits A_11_‐A_22_. b) Simulated shear deformation of the ceramic in (2–3) plane, and large shear strain exhibiting at the dotted area at the center. Shear deformations of c) two layers, d) four layers, e) six layers, and f) *n* layers (*n* > 40). g) Calculated tangential displacements *δy* of quasi‐d_34_ and d_15_ modes as a function of number of layers. Quasi‐d_34_ deformations for h) 20‐layer ceramic and i) 140‐layer ceramic. j) The deformation of d_15_ mode multilayer ceramic.

It is well known that piezoelectric d_34_ shear strain mode in piezoceramics means an electric field *E*_3_ induced shear strain *x*_4_ in (2–3) plane, as shown in Figure S1 in the Supporting Information. Unfortunately, the piezoelectric coefficient d_34_ is zero in natural PZT‐based piezoelectric ceramic materials. However, by designing an artificial ceramic structure based on OSPAU method, we may obtain the exotic macroscopic physical properties or parameters beyond nature. According to the idea, we designed a square multilayer ceramic actuator composed of four piezoceramic actuation units (PAUs) arranged in a (2 × 2) matrix (named as A_11_, A_12_, A_21_, and A_22_), as depicted in **Figure** **1**a. All four actuating units are polarized along 3‐direction, and the driving positive and negative voltages are applied along 3‐direction as well, meaning that the poling electric field and working voltages are sharing the same electrodes coated on the main surfaces of each ceramic layer, which is similar to a d_33_‐mode multilayer ceramic actuating unit. However, when the voltage applied to the two units along the diagonal (i–i) is opposite to that applied to another two units along the diagonal (ii–ii), the diagonal (i–i) will elongate (or contract), while the diagonal (ii–ii) will contract (or elongate) due to both d_33_ and d_31_ strains in each unit. The reversed deformations along two diagonals would cause an in‐plane distortion, i.e., the resultant effect of the reversed strains along two diagonals leads to the thickness shear strain in (2–3) plane of the square multilayer ceramic actuator. Therefore, this is a quais‐d_34_ shear mode according to the definition induced artificially.

Figure 1b illustrates the simulated (2–3) in‐plane quasi‐shear strain of the multilayered ceramic under asymmetric voltage drive with the bottom surface fixed, in which the color contour denotes the deformed shape (see Movie S1, Supporting Information). It is further observed that a much larger in‐plane shear deformation occurs at the center C than that at two ends of the square ceramic. As illustrated in Figure 1b, both tangential displacement *δy* along 2‐axis and vertical displacement *δz* along 3‐axis can be excited at the center point C by the quasi‐d_34_ mode. Using program voltages applied to the four subunits A_11_‐A_22_, the controllable actuations of the center part C either along diagonal (i–i) or (ii–ii) can be obtained, which is indispensable for a single‐mode piezoelectric actuator.

First, we theoretically estimated the effective piezoelectric strain coefficient d_34_ of the dotted area at the local point C, which could be expressed as

(1) $$\begin{array}{c} d_{34}=\frac{x_{4}}{E_{3}} \end{array}$$

where *x*_4_ is the quasi‐shear strain, and *E*_3_ is the applied working electric field.

Figure 1c further shows the enlarged in‐plane shear deformation of the dotted area with a length of *l*_c_ and a height of *h* to analyze the quasi‐shear strain *x*_4_ and corresponding (2–3) in‐plane shear angles. Assuming the center region C (dotted area) contains only four subunits, see Figure 1c, after reasonable simplification, the shear strain *x*_4_ can be computed as

(2) $$\begin{array}{c} \;x_{4}=\alpha_{23}\;+\alpha_{32}\approx \frac{\left|\delta z_{L^{\prime }}\right|}{l_{C}}+\frac{\left|\delta y_{L^{\prime }}\right|}{h} \end{array}$$

where *α*_23_ and *α*_32_ are the (2–3) in‐plane shear angles shown in Figure 1c; *δy*_L’_ and *δz*_L’_ are the displacements of the point L’ along 2‐direction and 3‐direction. In this way, we can derivate the effective piezoelectric strain coefficient d_34_ according to Equation (1).

Based on the analyses above, when the number of layers is relatively small, *x*_4_ remains constant, that means, the tangential displacement of the square ceramic increases linearly with the number of layers. As illustrated in Figure 1c–e, as the layer number *n* increases from 2 to 6, the tangential displacement increases correspondingly, but *x*_4_ remains almost unchanged. However, when adding more layers together, the shear‐bending effect occurs, as shown in Figure 1f, which would lead to the superposition effect.

Next, we used finite element method (FEM, by COMSOL Multiphysics) to simulate the quasi‐shear displacement *δy* of one multilayer square ceramic with different layers, in which the thickness of each layer was 250 µm. Assuming the electric field (1200 V mm^−1^) is applied to the subunits A_11_ and A_22_, the simulated quasi‐shear displacement *δy* is shown in Figure 1h. When the electric field is applied to the subunits A_12_ and A_21_, a reversed quasi‐shear displacement *δy* can be obtained.

It is further found that the quasi‐d_34_ mode induced tangential displacement *δy* at the center point C varies with the layer number *n* of the ceramic plate using the finite element method, as demonstrated in Figure 1g. When *n* is small, *δy* increases linearly with the increase of *n* (deformation simulation is shown in Figure 1h); however, when *n* is over 40 layers, the shear displacement *δy* exhibits a quadratic curving relationship due to shear‐bending deformation caused by the superposition effect of d_31_ and d_33_ strains (deformation simulation is shown in Figure 1i).

As a comparison, Figure 1g also illustrates the shear‐displacement simulation of a traditional d_15_‐mode multilayer structured actuator (with the same sizes as the quasi‐d_34_ mode actuator, as presented in Figure 1j) according to *δy*  =  *ntd*_15_ *E*_1_.^[^ ^31^ ^]^ Clearly, *δy* is linearly proportional to the layer number *n*. What's more, to avoid domain switching from length direction to thickness direction, the applied electric field *E* to each ceramic layer in the d_15_‐mode actuator is limited to only 300 V mm^−1^, which results in relatively lower shear‐displacement. As a contrast, quasi‐d_34_ shear‐mode actuator shows the advantage of larger displacement because the much higher electric field is permitted to be applied to itself; moreover, the additional shear‐bending effect also helps to produce larger displacement when the layer number *n* is high enough. Most importantly, the multilayer structure can be prepared through cofiring technology, which will further bring benefits of a low voltage drive, high‐integrity, and small volume. In Figure S4 in the Supporting Information, the vertical displacement comparison between quasi‐d_34_ mode and d_15_ mode would be further expounded.

**Figure 2:**
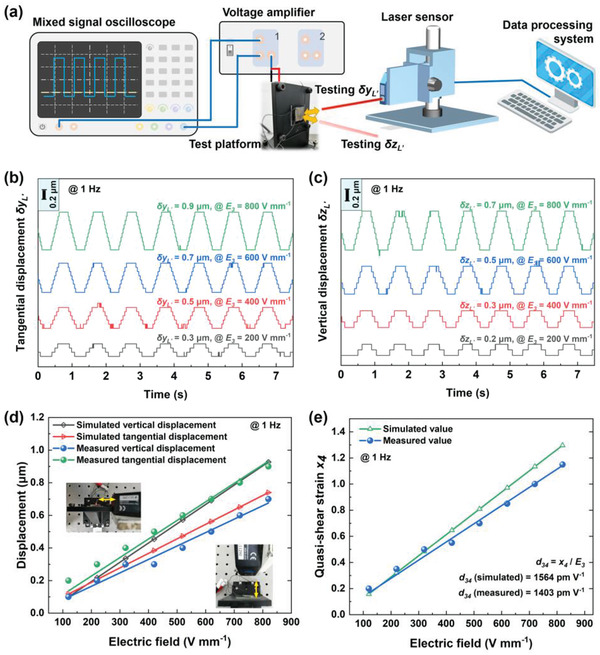
Experimental verification of the effective piezoelectric strain coefficient quasi‐d_34_ of the cofired eight‐layer quasi‐d_34_ shear‐mode ceramic. a) The graphical setup for measuring the tangential displacements *δy*_L’_ and the vertical displacements *δz*_L_ *_’_*. b) The tangential displacement responses and c) the vertical displacement responses under varying electric fields at 1 Hz. The simulated as well as measured d) displacement amplitudes and e) quasi‐shear strain *x*_4_.

In order to verify quasi‐d_34_ piezoelectric performances of the ordered structure experimentally, one cofired eight‐layer quasi‐d_34_ shear‐mode ceramic with dimensions of 15 mm × 15 mm × 2 mm was fabricated. Its detailed manufacturing process would be discussed in Experimental Section and Figure S2 in the Supporting Information. The center region C (see Figure 1b) was selected to investigate the effective piezoelectric strain coefficient quasi‐d_34_ of the cofired sample. Using the setup shown in **Figure** **2**a, the tangential displacements *δy*_L’_ and the vertical displacements *δz*_L_ *_’_* at point L′ (see Figure 1c) were measured using a laser sensor under a low‐frequency rectangular wave voltage drive. Figure 2b,c shows the dynamic response signals of tangential displacements and vertical displacements, respectively, under electric field drive in the range of 200 to 800 V mm^−1^. The measured displacement amplitudes in two directions are stable and repeatable, and they are also close to the simulated values obtained by finite element method, as depicted in Figure 2d. Using the measured tangential and vertical displacements, we can calculate the quasi‐shear strain *x*_4_ according to Equation (2). As presented in Figure 2e, it can be distinctly seen that the experimental results are in good agreement with the simulated results, though the measured values are slightly lower than the simulated ones. The rational difference is mainly on account of the fabrication processing, such as defective printed electrodes, incomplete polarization and nonideal boundary conditions etc. According to Equation (1), the effective piezoelectric strain coefficient quasi‐d_34_ can be further estimated to be 1403 pm V^−1^, which is quite close to the simulated value of 1564 pm V^−1^. Therefore, we artificially generate quasi‐d_34_ strain mode using the methodology of OSPAU in experiments, while it does not occur in natural piezoelectric ceramics.

### 2.2 Working Principle of Nonresonant Nanostep Actuator Operating in Quasi‐d_34_ Shear Mode

**Figure 3:**
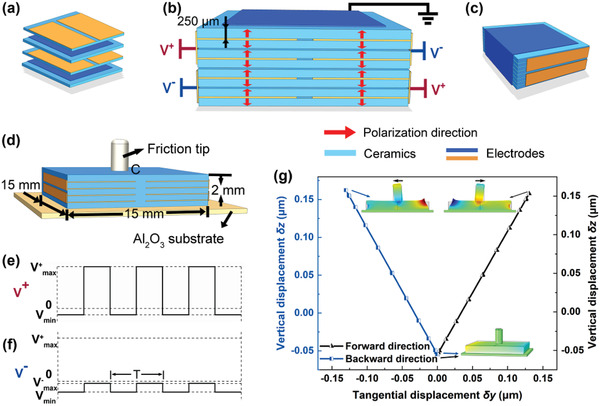
The devised (2 × 2) arrayed, cofired eight‐layer quasi‐d_34_ shear‐mode actuator and its operation principle. a) The laminating process with two types of internal electrodes. b) The detailed schematic means of the polarization and applied electric voltages. c) The integrated ceramic structure. d) The structure of the eight‐layer quasi‐d_34_ shear‐mode actuator. e) One programmable voltage *V* ^+^ applied to subunits along the diagonal (i–i) (or the diagonal (ii–ii), see Figure 1a). f) Another programmable voltage *V* ^−^ applied to driving units along the diagonal (ii–ii) (or the diagonal (i–i)). g) Simulated motion trajectories of the friction tip at the center point C of the actuator.

Based on the OSPAU method, one square multilayer ceramic actuator comprising of four head‐to‐head polarized subunits A_11_‐A_22_ in (2 × 2) array was designed, as depicted in **Figure** **3**a–d. A series of programmable low‐frequency rectangular‐wave voltages, *V* ^+^ and *V* ^−^, as shown in Figure 3e,f, were used to drive four subunits and produce unsymmetrical deformation (i.e., a quasi‐d_34_ shear mode) about its center line C‐C due to the synergistic effect of the anisotropic strain of the subunits. Therefore, it is rational to design a drive head at center part C for producing a controllable standing wave oscillation with a large amplitude (tangential displacement *δy*) there. The size of this multilayer ceramic is 15 mm × 15 mm × 2 mm, containing eight layers inside with the layer's thickness of 250 µm. For example, when the programmable voltage *V* ^+^ is applied to the subunits A_11_ and A_22_, while *V* ^−^ is applied to A_12_ and A_21_, the square multilayer ceramic plate will elongate along the diagonal (i–i) and shorten along the diagonal (ii–ii), producing shear motion in (2–3) plane. In this case, the standing wave oscillation generates a linear motion trajectory from the bottom left to the top right, and the motion trajectory of the drive head is depicted as the black line in Figure 3g. Similarly, when exchanging these two programmable voltage signals, the linear motion trajectory from the bottom right to the top left will be produced as depicted as the blue line in Figure 3g. Clearly, those two near orthogonal nonresonant standing wave oscillations can be respectively used to drive one contacting slider moving forward or backward via contacting friction force.

### 2.3 Actuation Experimental Results and Discussion

**Figure 4:**
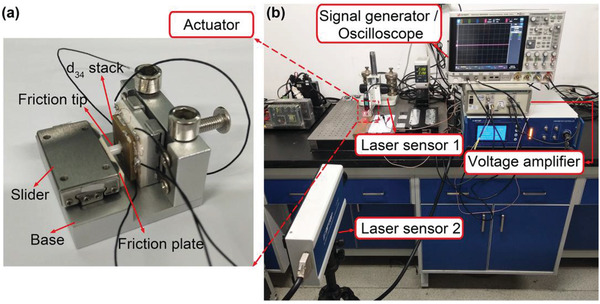
Nonresonant quasi‐d_34_ shear‐mode nanostep actuator and its experimental setup. a) The photograph of proposed nanostep actuator. b) The system consisting of a driving signal generation module, a voltage power amplifier, high precision displacement measurement modules, a digital oscilloscope, and a string‐pulley‐weight system.

To confirm the nano‐operation ability, a nanostep prototype composed of a quasi‐d_34_ shear‐mode cofired multilayer ceramic, an attached frictional coupling tip (zirconia (ZrO_2_)) at the center C, a slider with a frictional coupling ceramic plate (ZrO_2_ or silicon carbide (SiC)), and a base was built, as shown in **Figure** **4**a. The ceramic actuator was elastically pressed against the friction coupling plate of the slider via ZrO_2_ tip. Under the programmable control square‐waveform voltages, as shown in Figure 3e,f, the multilayer ceramic actuator produced a nonresonant quasi‐d_34_ shear‐mode oscillation (standing wave) at ZrO_2_ tip, driving the contacted slider in nanostep motion. The motion of the slider was measured by a laser sensor, as shown in Figure 4b. Additionally, a string‐pulley‐weight system was used for force‐loading capacity measurements.

**Figure 5:**
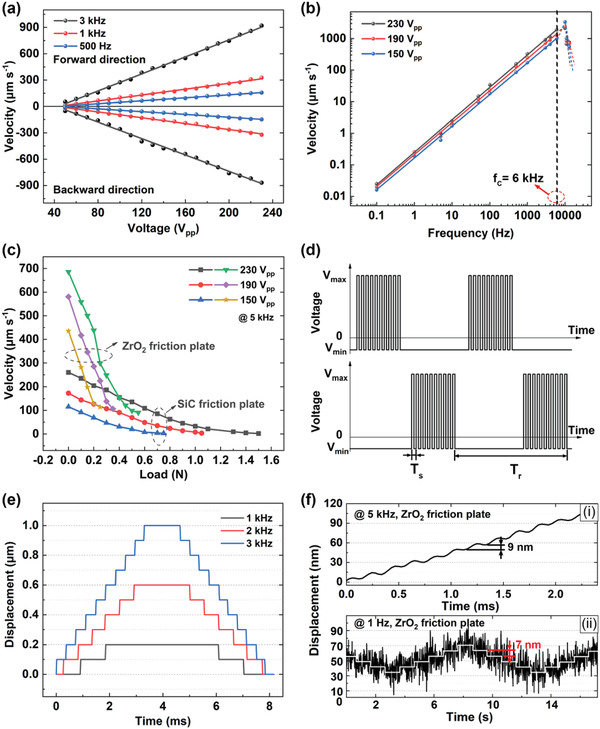
Experiments of nanostep actuation characteristics (using ZrO_2_ and SiC ceramics as the friction material). a) The output velocity of the slider as a function of driving voltage at 500 Hz, 1 kHz, and 3 kHz. b) The relationship between the velocity and working frequency under diverse voltages. c) The driving voltage and load‐carrying capacity test via ZrO_2_ and SiC frictional plates at 5 kHz. d) Voltage driving signals for bidirectional motions. e) The bidirectional step performances of the actuator. f) The displacement resolution of the actuator at i) 5 kHz and ii) 1 Hz.

**Figure** **5**a shows the measured moving velocities of the slider in two directions as a function of the driving voltage at different frequencies of 500 Hz, 1 kHz, and 3 kHz in a nonresonant state. As can be seen from Figure 5a, the starting voltage for driving the slider is about 50 V_pp_. When the applied voltage exceeds 50 V_pp_, the moving velocity of the slider goes almost linearly with the increase of the applied voltage, meaning that the moving or step displacement speed of the slider can be linearly controlled by the input voltage. Moreover, it is further observed from Figure 5a that the produced moving velocity in both forward and backward directions are quite symmetrical, meaning two symmetric d_34_ modes are generated.

Figure 5b describes the frequency‐dependent moving velocities under varying voltages. It is clear that the actuator can work within a wide frequency range from 0.1 Hz to 6 kHz in a nonresonant state, where the moving velocity is well proportional to the operating frequency and reaches its maximum value of 1.89 mm s^−1^ at 6 kHz. The moving velocity *v* as a function of operating frequency *f* and quasi‐shear displacement Δ*y* can be given as

(3) $$\begin{array}{c} v\;=f\cdot \Delta y\left(V\right) \end{array}$$

where Δ*y*(*V*) is a function of the applied voltage. Equation (3) implies that each step produced by the actuator is independent of the operating frequency. When the operating frequency is higher than 6 kHz, the actuator exhibits abnormal speed behavior with a drop. This phenomenon may be related to the power amplifier because it cannot afford a powerful current at high frequency to drive high‐capacitance multilayer piezo‐actuator.

With a pulley‐weight method, we could further obtain the load and velocity relationship. Figure 5c illustrates the measured moving velocity of the slider as a function of the mechanical load under varying voltage at 5 kHz, and it is found that the maximum mechanical load of the miniature actuator is about 0.55 N via ZrO_2_ friction plate and 1.5 N via SiC friction plate, respectively. The difference in mechanical load is obvious, as the friction coefficients of the diverse friction pairs would definitely lead to different driving force. What's more, the velocity decreases almost linearly with the increase of the load.

To acquire the bidirectional step displacement response, two intermittent square‐wave voltage signals with a phase difference of 180°, as shown in Figure 5d, were applied to the actuating units A_11_ and A_22_, and A_12_ and A_21_, respectively. Note that in Figure 5d, *T*_s_ refers to the step cycle (i.e., the slider moves one step in each step cycle), and *T*_r_ represents the period of bidirectional step displacements (i.e., the slider moves ten steps forward, and then, ten steps backward). Also, to obtain more stably motion, an idle time between two intermittent square‐wave voltage signals was set, which determined dead time between forward and backward step movement. Figure 5e shows the measured step displacement versus time of the actuator operating at different step frequency *f*_s_. It is clearly seen that the actuator under a given voltage produces nearly the same step displacement of 0.1 µm for the three operating frequencies, which also verifies the independence between the stepping displacement and the operating frequency in working frequency range, as also deduced from Figure 5b.

Finally, the step resolution of the nanostep actuator under varying voltage amplitudes and frequencies was evaluated by using a laser sensor. The stepwise excitation signals are presented in Figure 3e,f. The minimum displacement of 9 nm was found under the voltage of 50 V_pp_ at the frequency of 5 kHz, i.e., the displacement resolution of nanostep actuator in open‐loop control is about 9 nm as shown in Figure 5f‐i. While working at the frequency of 1 Hz, the measured displacement resolution is about 7 nm although low‐frequency environment noise also increases, as shown in Figure 5f‐ii. Superhigh resolution of the actuator should be attributed to the nonresonant working mechanism of d_34_ quasi‐shear mode. Implementation of high accuracy positioning at the scale of nanometer will bring great benefits to future biomedical engineering field, because of its potential to be utilized as most precise finger or knife actuator in surgical robots.

**Table** **1** summaries the main actuation performances of the nonresonant nanostep actuator in this work and previously reported piezoelectric actuators. It is clearly seen that the presented quasi‐d_34_ shear‐mode actuator shows great superiority in the displacement resolution, and it can also run at the velocity of millimeter per second. What's more, the proposed nanostep actuator is much simpler in structure and driving circuit compared with the inertia actuators and the inchworm actuators operating in stepping principle. Besides, since piezoelectric actuators with a PID feedback (closed‐loop control) could achieve much higher displacement resolution,^[^ ^17^, ^26^ ^]^ it is rational to assume that the nanostep actuator will produce much higher positioning accuracy if a closed‐loop feedback control is adopted.

**Table 1:** Main performance comparison between the proposed nanostep actuator and previously reported piezoelectric actuators

Parameters	This work	The ultrasonic actuator by Kim et al.^[^ ^17^ ^]^	The planar actuator by Deng et al.^[^ ^23^ ^]^	The inertia actuator by Zeng et al.^[^ ^24^ ^]^	The inchworm actuator by Moon et al.^[^ ^26^ ^]^
Working state	Nonresonant	Resonant	Nonresonant	Nonresonant	Nonresonant
Displacement resolution [nm]	7	20	16	20	50
Maximum speed [mm s^−1^]	1.89	450	0.3	16.87	10.2
Control method	Open‐loop	Closed‐loop	Open‐loop	Open‐loop	Closed‐loop

## 3 Conclusion

In summary, according to the methodology of ordered structure with function units, we developed a piezoelectric actuator based on (2 × 2) arrayed, cofired multilayer piezoceramic actuation units, which operates in artificially designed quasi‐d_34_ shear strain mode for realizing continual nanostep actuation. The obtained effective quasi‐d_34_ value from the cofired multilayer ceramic is as high as 1403 pm V^−1^, while it is zero in natural piezoelectric ceramics. The proposed actuator works in nonresonant quasi‐d_34_ shear strain mode in the frequency range of 0.1 Hz to 6 kHz and produces continuously bidirectional step motions from nano‐ to macro‐scale with almost unlimited stroke (as long as its slider is long enough). Measurements showed that under a single‐phase square‐wave voltage of 230 V_pp_, the generated maximum step motion velocity and the mechanical load were 1.89 mm s^−1^ and 1.5 N, respectively. Furthermore, a superhigh resolution of 7 nm in open‐loop control was observed, which clearly confirms the nanoscale positioning ability, demonstrating promise for applications such as most precise finger or knife actuation of future minimally invasive surgical robots in biomedical engineering field. The proposed method in this work is also of great guide significant for designing future actuators based on artificially designed, arrayed piezoceramic structures.

## 4 Experimental Section

##### Finite Element Simulation

All simulations on electric field induced piezoelectric deformations and displacement character values of multilayer ceramic were carried out by using the module of piezoelectric device in COMSOL Multiphysics. The geometrical models of the actuator were built according to the design methodology of OSPAU; the thickness of each layer in multilayer ceramic was supposed to be 250 µm for all work. The ceramic material PZT‐5H was the default material in the built‐in material libraries, while its d_33_, d_31_, and d_15_ coefficients were altered to 650, −300, 780 pm V^−1^ according to the commercialized PZT‐5H used. The bottom of the structural was set as fixed as mechanic boundary condition as shown in Figure 1a, while four subunits of the multilayer ceramic structure were set as the head‐to‐head polarization by using the base vector systems. With voltages, charges and other parameters set on the electrostatic interface, the displacements and the motion trajectories were simulated according to the static and dynamic conditions, respectively. Note that to avoid domain switching, the applied electric field is limited to only 300 V mm^−1^ when the applied voltage direction is antiparallel to the polarization direction of the subunit. The particular diagram of the preparation processes and the characterization are shown in Figures S2 and S3 in the Supporting Information.

##### Sample Preparation

The piezoelectric ceramic powders (PZT‐5H) were offered by BaoDing HongSheng Acoustics Electron Apparatus Co. Ltd. (Baoding, China) with the typical properties listed in Table S1 in the Supporting Information. Homogenous slurry having 55% solid loading of PZT‐5H particles with polyvinyl alcohol (PVA) and ethyl acetate as binder and solvent respectively was subjected to ball milling in nylon bottles for 24 h. The slurry was then made into ceramic films by tape casting method, and next, according to subunit design, the Ag‐Pd high‐temperature electrode patterns were printed on the ceramic films by screen printing method. Then the ceramic films were cut into a specified size and then laminated together. After dumping at 600 °C, the laminated ceramic was sintered at 960 °C for 3 h for crystallization to form a cofired multilayer structure. It should be noted that one pair of ceramic layers were respectively superimposed on the upper and lower sides for electric insulation. Next, external silver electrodes were coated on four side faces of the multilayer structure for welding wires. Finally, ceramic layers (each layer is about 250 µm thick) inside the multilayer structure were head‐to‐head polarized in thickness direction in silicon oil bath under a DC electric voltage of 2 kV mm^−1^ at 120 °C for 10 min.

##### Parameter Measurement

The piezoelectric strain coefficient was measured with quasi‐static piezoelectric meter (ZJ‐3D, Institute of Acoustics). The cross‐sectional EDS images were observed with scanning electron microscopy (JSM‐IT300, Jeol). The acquired square‐wave voltage signals were produced and monitored using a mixed signal oscilloscope (MSOX4024A, Keysight) after amplifying with a high‐voltage amplifier (2350, Tegam). Then the actuation characteristics were measured using the high‐precision laser sensors (LK‐G30, Keyence; LV‐S01, Sunny; and LY1000, LeiCe).

## Conflict of Interest

The authors declare no conflict of interest.

## Supporting information

Supporting Information

Supplemental Movie 1
