# Supplementary material for: Designing Ordered Structure with Piezoceramic Actuation Units (OSPAU) for Generating Continual Nanostep Motion
Source: Adv Sci (Weinh). 2020 Jul 2;7(16):2001155. doi: 10.1002/advs.202001155 (PMC7435238; doi:10.1002/advs.202001155)
Supplement: Supplementary file 1 — Supporting Information [file ADVS-7-2001155-s001.pdf]

Copyright WILEY-VCH Verlag GmbH & Co. KGaA, 69469 Weinheim, Germany, 2020.

Supporting Information

Designing Ordered Structure with Piezoceramic Actuation Units (OSPAU) for Generating Continual Nano-Step Motion

*Zhanmiao Li, Xiangyu Gao, Jikun Yang, Xudong Xin, Xingyu Yi, Lang Bian, and Shuxiang Dong**

Supporting Information

Designing Ordered Structure with Piezoceramic Actuation Units (OSPAU) for Generating Continual Nano-Step Motion

*Zhanmiao Li, Xiangyu Gao, Jikun Yang, Xudong Xin, Xingyu Yi, Lang Bian, and Shuxiang Dong**

Section S1. Design and fabrication of the novel co-fired multilayer quasi-d_{34} shear-mode actuator

When one piezoelectric material is polarized along 3-direction and the induced deformation under the applied electric field along 3-direction is in (2-3) plane, this strain is defined as d_{34} shear mode according to the mode definition in piezoelectric crystals, as shown in **Figure S1**. While the strain tensor x_4 is defined as

$$x_4 = \theta_1 + \theta_2 \tag{S1}$$

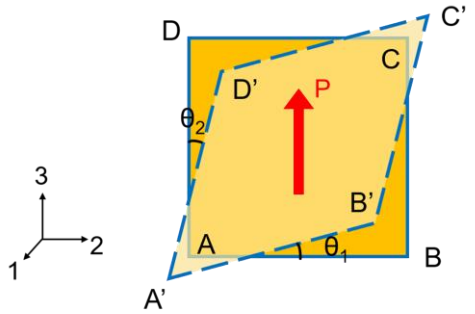

Figure S1. Scheme of general d_{34} shear mode deformation.

The co-fired multilayer structure is an effective method to obtain the miniaturized and integrated devices. Meanwhile, its advantages of low driving voltages and multi-functionalization render it widely used in the field of precision positioning, such as actuators, ultrasonic transducers and other devices from decades ago. Shear-mode configuration is inevitable for the continuous operation of the piezoelectric actuators. However, the traditional multilayer shear-mode piezoelectric actuators cannot be fabricated by co-fired method because of the conflict between the poling direction and applied electric field direction. To solve this problem, we designed and fabricated a novel co-fired multilayer quasi-d_{34} shear-mode actuator based on the mythology of ordered structure with piezoceramic actuation units, which has been disclosed in Figure 3a-d and Experimental Section.

Table S1 illustrates the main properties of the piezoelectric ceramic powders (PZT-5H) we used in the sample preparation processes.

Table S1. Piezoelectric and dielectric properties of PZT-5H.

ε_r	d_{33} (10^{-12} C N^{-1})	d_{31} (10^{-12} C N^{-1})	d_{15} (10^{-12} C N^{-1})	k_p	Q_m	tanδ
3200	650	-300	780	0.68	70	2%

After the elaborate design, we began the multilayer fabrication with ball milling ceramic slurry, which is the ingredient for ceramic films using tape casting method as shown in **Figure S2**a. The electrode patterns were then printed on the films by screen printing method, as depicted in Figure S2b-i and Figure S2b-ii. After laminating, dumping and sintering, the

multilayer ceramic formed a crystallized structure (see Figure S2c-e). The prepared sample of co-fired eight-layer ceramic actuator with dimensions of 15 mm × 15 mm × 2 mm is shown in Figure S2f.

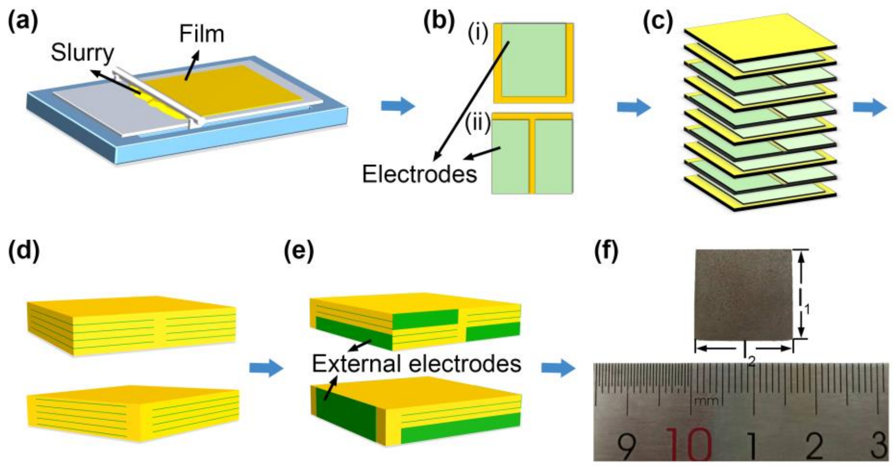

Figure S2. Preparation of co-fired multilayer ceramic with four subunits array. Processes of the co-fired multilayer ceramic: a) tape casting, b) screen printing, c) laminating, d) co-firing, and e) coating external electrodes. f) Prepared d_{34} co-fired eight-layer ceramic.

Figure S3 shows the EDS images of some main elements on the cross section of the multilayer structure, and it is clear that there are five full electrodes and four two-part-segmented electrodes as expected, confirming the integrity of preparation process.

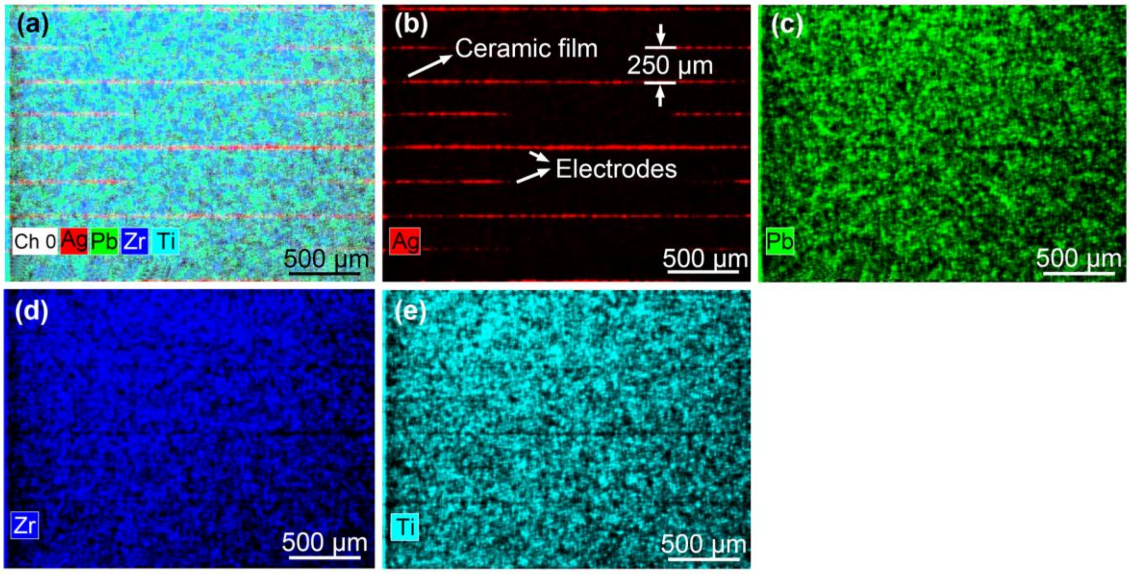

Figure S3. Cross-sectional EDS images of the co-fired eight-layer ceramic: a) the combined elements, b) silver (Ag), c) lead (Pb), d) zirconium (Zr), and e) titanium (Ti).

Section S2. Deformation comparison between quasi-d_{34} mode and d_{15} mode when increasing layers

Excitation of two orthogonal vibrations is necessary for producing two-way motions of the piezoelectric actuators. The tangential displacement comparison between quasi-d_{34} mode and d_{15} mode has been discussed in Figure 1g-j, and it has been proved that quasi-d_{34} mode has superposition effect. Setting each film layer to be 250 μm thick, the simulated vertical displacements δz for these two shear modes as a function of the number of layers are illustrated in **Figure S4**a. We can clearly see that the vertical displacement of d_{34} shear mode is proportional to the layer number *n*, and it reaches 20 μm when *n* is over 180 layers. What's more, no matter how *n* raises, the vertical displacements for the d_{15} mode are very small that can be negligible. This phenomenon indicates that quasi-d_{34} mode has an absolute advantage over d_{15} mode in terms of independent actuation, in addition to the capacity for using co-fired method. The vivid deformations of these two shear modes with 180 layers are depicted in Figure S4b,c for better comprehension.

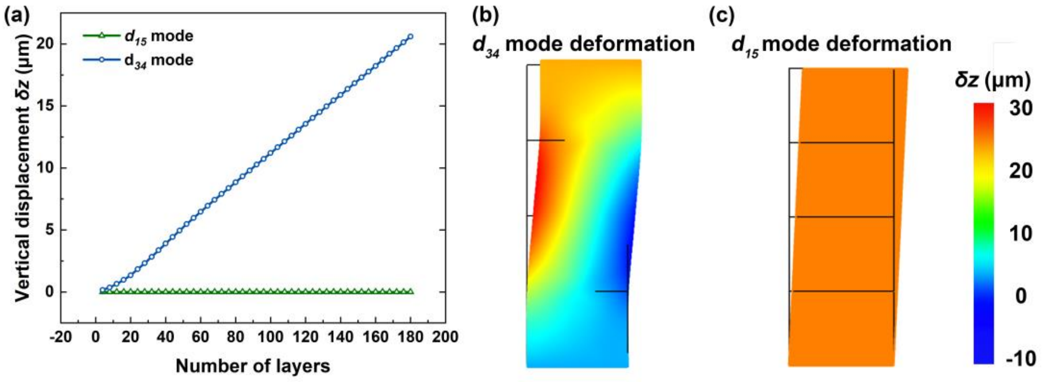

Figure S4. The vertical displacements δz of quasi-d_{34} shear mode and d_{15} shear mode with increasing layers. a) Calculated vertical displacements δz of quasi-d_{34} and d_{15} modes as a

function of number of layers. Graphical deformations of b) quasi-d_{34} mode and c) d_{15} mode for 180-layer ceramic where the legend color represents the vertical displacement.
